# Impact of cross-disorder polygenic risk on frontal brain activation with specific effect of schizophrenia risk

**DOI:** 10.1016/j.schres.2014.10.046

**Published:** 2015-02

**Authors:** Heather C. Whalley, Lynsey Hall, Liana Romaniuk, Alix Macdonald, Stephen M. Lawrie, Jessika E. Sussmann, Andrew M. McIntosh

**Affiliations:** Division of Psychiatry, University of Edinburgh, Edinburgh, UK

**Keywords:** Polygenic, Schizophrenia, Frontal cortex, fMRI

## Abstract

Evidence suggests that there is shared genetic aetiology across the major psychiatric disorders conferred by additive effects of many common variants. Measuring their joint effects on brain function may identify common neural risk mechanisms. We investigated the effects of a cross-disorder polygenic risk score (PGRS), based on additive effects of genetic susceptibility to the five major psychiatric disorders, on brain activation during performance of a language-based executive task. We examined this relationship in healthy individuals with (n = 82) and without (n = 57) a family history of bipolar disorder to determine whether this effect was additive or interactive dependent on the presence of family history. We demonstrate a significant interaction for polygenic loading × group in left lateral frontal cortex (BA9, BA6). Further examination indicated that this was driven by a significant positive correlation in those without a family history (i.e. healthy unrelated volunteers), with no significant relationships in the familial group. We then examined the effect of the individual diagnoses contributing to the PGRS to determine evidence of disorder-specificity. We found a significant association with the schizophrenia polygenic score only, with no other significant relationships. These findings indicate differences in left lateral frontal brain activation in association with increased cross-disorder PGRS in individuals without a family history of psychiatric illness. Lack of effects in the familial group may reflect epistatic effects, shared environmental influences or effects not captured by the PGRS. The specific relationship with loading for schizophrenia is notably consistent with frontal cortical inefficiency as a circumscribed phenotype of psychotic disorders.

## 1 Introduction

Current diagnostic criteria in psychiatry are based around symptom patterns and course of illness, however, no symptom is uniquely associated with an individual condition, and symptoms vary between people with the same diagnosis. Psychosis, mood instability, and cognitive impairments for example are observed across multiple diagnoses. There is also considerable overlap in genetic contributions, as well as commonalities in implicated brain networks, for example the prefrontal cortex and medial temporal lobes (Phillips et al., 2003; Shaw and Rabin, 2009; Dickstein et al., 2013; Hong Lee et al., 2013). There is an increasing uncertainty therefore over the degree to which current diagnostic criteria define biologically-valid distinct entities, or whether common mechanisms contribute to multiple conditions or cross-disorder phenotypes.

To address such issues, previous imaging studies have employed a dimensional approach, by examining the neurobiology of specific symptoms crossing diagnostic boundaries. These have included individuals with, or at increased risk of, schizophrenia with and without mood symptoms (Whalley et al., 2008; Simon et al., 2010; Tomasino et al., 2011; Barbour et al., 2012), and patients with mood disorder with and without psychotic features (Sommer et al., 2007; Khadka et al., 2013). Although literature is limited, evidence suggests alterations in medial temporal lobe and limbic structures in association with mood-related symptoms across disorders (Tomasino et al., 2011), and alterations in lateral prefrontal functioning in association with psychosis, also trans-diagnostically (Anticevic et al., 2013).

Genetic imaging studies have also examined the impact of shared genetic risk on underlying neurobiology. Previous studies have investigated the effects on neurobiology of individual SNPs identified as potential risk markers for illness within and across diagnostic groups (Mechelli et al., 2008; Chakirova et al., 2011; Papagni et al., 2011; Prata et al., 2011; Whalley et al., 2012a,b). Current evidence however suggests that for psychiatric disorders a substantial proportion of the heritability is explained by a polygenic component. We previously used the polygenic approach to demonstrate increased activation of mood-related limbic regions in association with increased polygenic loading for bipolar disorder (Whalley et al., 2012a,b, 2013).

One recent study has used genetic strategies to explore *shared* genetic architecture across the 5 major psychiatric disorders using Psychiatric Genomics Consortium data (Smoller et al., 2013). The authors identified shared genetic effects between Attention Deficit Hyperactivity Disorder (ADHD), Autism (Aut), Bipolar disorder (BD), Major Depressive Disorder (MDD) and Schizophrenia (SCZ), in 33,332 cases and 27,888 controls (Smoller et al., 2013), firstly by examining effects of shared GWAS hits for BD and SCZ, and then by generating cross-disorder polygenic risk scores (PGRSs) to examine a broader set of common variants. This cross-disorder PGRS is likely to account for an even greater proportion of overall risk than for single disorder PGRS and allows examination of processes involved in enhanced risk across diagnostic groups (Smoller et al., 2013).

In the current study we examine the neural effects of this broader set of common variants on brain activation in regions previously associated with the 5 major psychiatric disorders, namely the prefrontal cortex and medial temporal lobe structures (Phillips et al., 2003; Shaw and Rabin, 2009; Dickstein et al., 2013). We also sought to test whether there was an additive or interactive effect of family history on the effect of PGRS on neural activation by examining groups with and without a family history of mood disorder. The paradigm, a language-based executive function task, was chosen as it had previously been shown to differentiate psychiatric patients, and those at increased familial risk, from healthy controls in these regions (McIntosh et al., 2008a,b; Whalley et al., 2011). Moreover, it probes frontal neuropsychological deficits in executive function, verbal initiation and verbal fluency seen across a range of psychiatric disorders (Clark et al., 2000; Arts et al., 2008; Booth and Happe, 2010).

We were also interested in examining whether there was any evidence for disease-specific brain activation associations by deconstructing the components of the cross-disorder PGRS into diagnosis-specific sub-scores (Smoller et al., 2013). Based on neuroimaging evidence described, we hypothesised that there would be abnormal frontal activation in association with increased loading for schizophrenia, and increased activation of medial temporal regions in association with mood disorder.

## 2 Methods

### 2.1 Study population

Individuals at high genetic risk of bipolar disorder I (BDI), because of a close family history of the disorder, and control subjects with no family history were recruited as part of the Scottish Bipolar Family Study (Sprooten et al., 2011; Whalley et al., 2011). Caseloads of psychiatrists across Scotland were searched for individuals diagnosed with BDI. Diagnoses were confirmed with the Structural Clinical Interview for DSM-IV-TR Axis I Disorders (SCID-I) (First et al., 2002) or the symptom checklist of Operational Criteria (OPCRIT) (McGuffin et al., 1991). Subjects with BD were asked to identify a first or second-degree relative (between 16–25 years) not suffering from the disorder. These unaffected individuals were invited to participate in this study provided they had at least one first degree, or two second degree relatives with BDI. Controls with no personal history of BD or family history of a mood disorder in first-degree relatives were identified from the personal contacts of the bipolar high-risk subjects. Exclusion criteria for all groups included a personal history of major depression, mania or hypomania, psychosis, substance dependence, an IQ < 70 or clinical diagnosis of learning disability, or any major neurological disorder or history of head injury that included loss of consciousness, and any contraindications to MRI. A total of 82 bipolar high-risk and 57 controls provided suitable fMRI data and genetic information. All participants provided written informed consent and the study was approved by the multi-centre research ethics committee for Scotland. All participants included in the current study were unrelated.

### 2.2 Genotyping and derivation of polygenic scores

Genomic DNA was extracted from venous blood. Genotyping was conducted at the Wellcome Trust Clinical Research Facility, Edinburgh, United Kingdom (www.wtcrf.ed.ac.uk) using the Illumina OmniExpress 730K SNP array. PGRS analyses were performed in PLINK (Purcell et al., 2007) using imputed genotype data. Imputation was performed in accordance with the 1000 Genomes Project Protocol SNPs with an imputation quality score of r2 > 0.3 retained for analysis. Methods for creating PGRS are described elsewhere (Purcell et al., 2009). Summary statistics from the PGC GWAS Cross Disorder group (33,332 cases and 27,888 controls) were used as the training set to create cross-disorder PGRS for our samples (Smoller et al., 2013). Our primary analyses concerned those SNPs from the PGC data that met a significance level of p = .5 or less as previously described (Purcell et al., 2009; Whalley et al., 2012a,b, 2013), further details in Supplementary material.

### 2.3 Clinical assessments

All participants were interviewed by one of the two experienced psychiatrists (AMM, JES) using the SCID (First et al., 2002) to confirm the absence of any lifetime axis I disorders. Current symptoms were rated using the Young Mania Rating Scale (YMRS) (Young et al., 1978), Hamilton Rating Scale for Depression (HAM-D) (Hamilton, 1960), and the positive and negative syndrome scale (PANSS) (Kay et al., 1987).

### 2.4 Experimental paradigm

Subjects performed the verbal initiation section of the Hayling Sentence Completion Test (HSCT) (Burgess and Shallice, 1997) in the scanner (Whalley et al., 2004). This is an extension of the verbal fluency task and considered a test of executive function. Briefly, subjects were shown sentences with the last word missing and asked to think of an appropriate word to complete the sentence and press a button when they had done so. The task has four levels of difficulty, according to the range of suitable completion words suggested by the sentence context. This allowed a standard subtraction analysis (sentence completion versus baseline) and a parametric analysis (examining increasing activation with increasing task difficulty). Sentences were presented in blocks of fixed difficulty. The order of the blocks was pseudo-random, and each block was repeated four times using different sentences. Immediately after scanning, subjects were given the same sequence of sentences on paper and requested to complete each sentence with the word they first thought of in the scanner. ‘Word appropriateness’ scores were determined from the word frequency list of sentence completion norms (Bloom and Fischler, 1980).

### 2.5 Image processing and analysis

Scanning procedure details are contained in Supplementary material. EPI and T1 images were reconstructed into nifti format (Mayo Foundation, Rochester, MN, USA) using DICOM convert functions in SPM5 (Statistical Parametric Mapping: The Wellcome Department of Cognitive Neurology and collaborators, Institute of Neurology, London) running in Matlab (The MathWorks, Natick, MA, USA). Images were pre-processed using standard protocols in SPM5. All EPI images were realigned to the mean volume in the series. Functional images were then normalised according to standard co-registration procedures using the individual's structural scan. Finally, all realigned and normalised images were smoothed with an 8 × 8 × 8 mm full width half maximum (FWHM) Gaussian filter.

First-level analysis was performed using the general linear model. At the individual subject level the data was modelled with four conditions corresponding to the four difficulty levels each modelled by a boxcar convolved with a synthetic haemodynamic response function. Estimates of the subject's movement were entered as ‘covariates of no interest’. The participant's data was filtered in the time domain using high pass filter (128 s cut-off) and serial correlations were accounted for by using the first order autoregressive model. Contrasts were constructed to examine all four levels of sentence completion difficulty versus baseline, and areas of increasing activation with increasing task difficulty (the parametric contrast).

### 2.6 Second-level analysis

For each contrast of interest, one contrast image per subject was entered into a second-level random effects analysis. The cross-disorder PGRS for each individual was entered into a full factorial model as a single regressor per group. The four multidimensional scaling (MDS) factors were entered as additional ‘nuisance’ covariates to control for population stratification, along with age and IQ (NART).

Statistical maps were thresholded at a level of p < 0.001 (uncorrected). Regions were considered significant at a cluster level of p < 0.05, corrected for multiple comparisons. All coordinates are quoted in Montreal Neurological Institute (MNI) convention (http://www.mni.mcgill.ca) and images are overlaid onto standard brain in MNI space using Mango software package (http://ric.uthscsa.edu/mango). Regions of interest included frontal brain regions (whole brain level) and amygdala and hippocampus using small volume corrections (svc's) created using the WFU PickAtlas (Tzourio-Mazoyer et al., 2002; Maldjian et al., 2003).

## 3 Results

### 3.1 Demographic, clinical, temperament and behavioural measures

There were no significant differences between the groups in terms of age, gender, or NART IQ (Table 1). The groups differed in terms of the cross-disorder PGRS (p = 0.05), the disorder-specific sub-scores for SCZ (p = 0.03), and at the trend level for BD (p = 0.06) where the familial group scored significantly higher than those without familial risk.

Groups also differed on measures of depression (from the HAM-D, p = 0.02), and PANSS positive scores (p = 0.02), with the familial group scoring highest. There were no significant group differences in the within-scanner measures of reaction time or word appropriateness. Both groups demonstrated the typical gradation in these behavioural measures according to task difficulty (Whalley et al., 2011).

### 3.2 Task-related brain activation

All subjects also demonstrated the expected patterns of brain activation indicating that subjects were performing the task appropriately in the scanner (Whalley et al., 2004; McIntosh et al., 2008a,b; Whalley et al., 2011). Regions activated for the sentence completion versus baseline contrast included the left medial and lateral prefrontal regions, left lateral temporal cortex, sub-cortical structures, left lateral parietal cortex, occipital lobes bilaterally, and right cerebellum, see Supplementary Fig. 1.

### 3.3 Effects of cross-disorder PGRS on neural activation

**Fig. 1:**
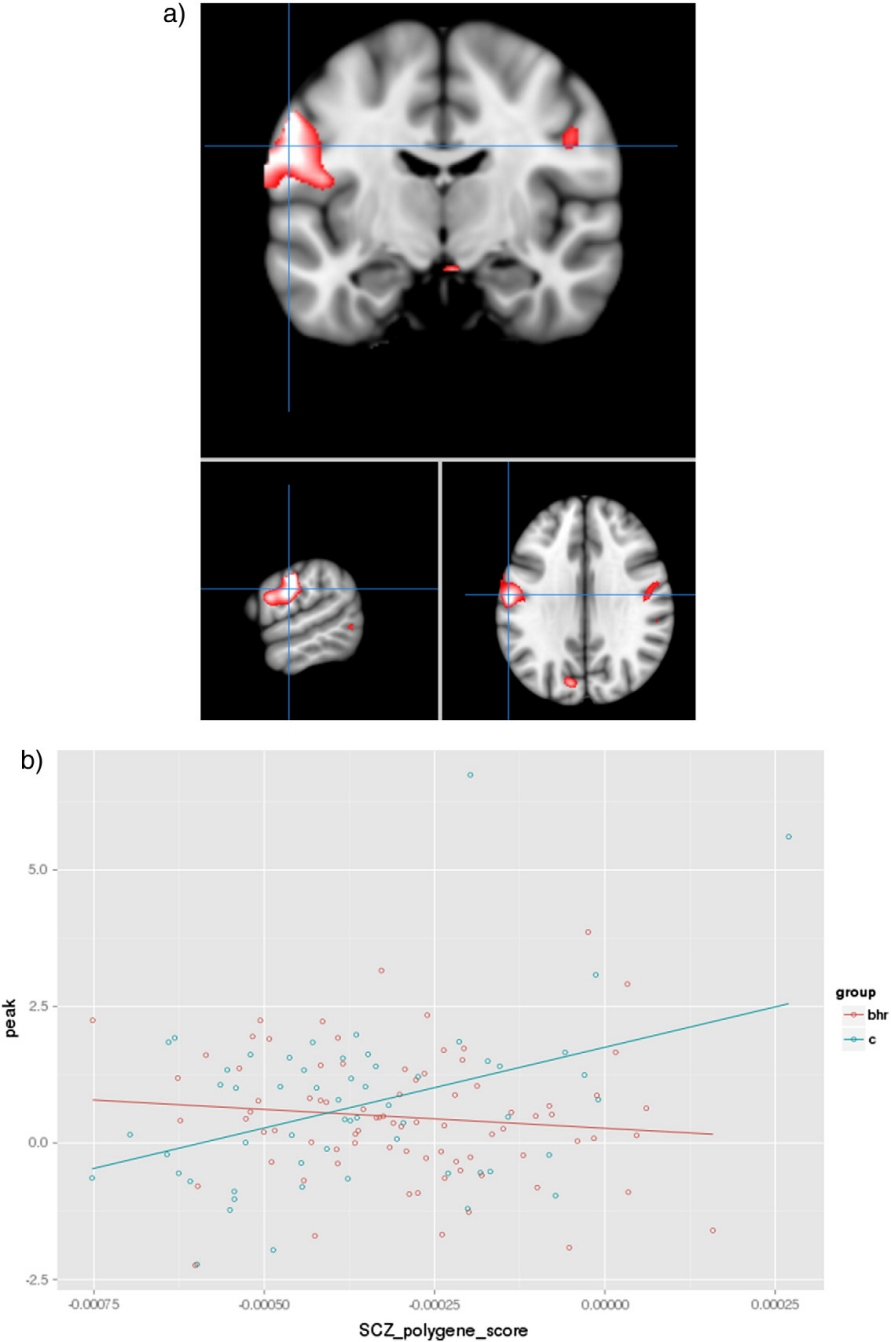
Interaction between groups for cross disorder PGRS in frontal cortex. a, Depicts significant interaction between individuals with and without family history of mood disorder in the frontal cortex. Images are overlaid onto standard brain in MNI space using Mango software package (http://ric.uthscsa.edu/mango). Map represents T-statistic images thresholded equivalent to p uncorrected = 0.001, see Methods for further details. b, Presents scatter plot of peak of activation versus PGRS for schizophrenia in both groups, blue—controls (without family history), red—bipolar high risk (with family history).

For sentence completion versus baseline, there were no significant relationships across the groups between the cross-disorder PGRS and brain activation. There was however a statistically significant cross-disorder PGRS × group interaction in a large cluster encompassing the left inferior frontal gyrus, precentral and postcentral gyri (see Fig. 1a,b: Brodmann areas 9 and 6; p < 0.001, K_E_ = 817, Z = 4.63 [− 58, − 14, 38]). Examining data within groups separately indicated that this was driven by a significant positive effect in those without family history (p < 0.001, K_E_ = 1210, Z = 4.91 [− 58, − 16, 28]), with no significant effects in the familial group. There were no significant findings for the parametric contrast, and no significant findings for either contrast in medial temporal lobe regions.

Data for the peak of this main interaction was then extracted to explore the relationship with the composite sub-scores (within the family history negative group) to determine if one of the diagnoses was driving this main effect. The only individual diagnosis that demonstrated a significant correlation was the sub-score for schizophrenia, both when determined separately and whilst controlling for the other diagnoses (p < 0.01 in each case, Table 2).

### 3.4 Effects of potential confounders

Neither the cross-disorder PGRS, nor the schizophrenia PGRS sub-score correlated with NART IQ, nor any of the clinical measures, either across or within groups. We also examined the relationship between the MDS components and the extracted values from the peak cluster. There were no significant correlations between these measures indicating that the above findings were not confounded by population stratification.

## 4 Discussion

Here we report an impact of cumulative genetic risk for the five major psychiatric disorders on brain activation in frontal regions. This was observed in the group without a family history for mood disorder, where increasing cross-disorder PGRS was associated with increased frontal activation. Examination of the contributing sub-scores suggested that this effect was specific to elevated risk for schizophrenia. The result was not associated with population stratification, age, or IQ, and was not confounded by illness or medication effects.

Our findings indicated that the relationship between frontal activation and PGRS was strongest for the association with the schizophrenia sub-score. We note that whilst this finding is consistent with a specific effect of genetic risk for schizophrenia on brain function, other explanations should be considered. For example, this may be due to the larger discovery GWAS sample in schizophrenia and/or the greater proportion of phenotypic variance explained by the PGRS in schizophrenia compared to other psychiatric disorders. However, the finding is highly consistent with observations that deficits in frontal cognition are a core feature of schizophrenia (Simonsen et al., 2011). Similarly, imaging studies consistently report altered frontal activation in patients with the disorder during performance of executive function tasks (Manoach, 2003; Glahn et al., 2005). It is also highly consistent with a previous report suggesting that elevated polygenic risk for schizophrenia (based on ~ 600 SNPs) correlated significantly with neural ‘inefficiency’ or ‘compensation’ in the left lateral prefrontal cortex during an executive task (Walton et al., 2014). The current work extends this to suggest that this effect is specific, or has a stronger relationship to schizophrenia risk, rather than being related to a generalised elevated risk to the other major psychiatric disorders. Further studies in different patient populations could explore whether this relationship extends to other disorders and reflects a true trans-diagnostic effect.

As predicted, the cross-disorder PGRS, along with SCZ and BD sub-scores, was higher in those with a family history of mood disorder versus those without. The imaging findings suggest, however, that there was a group × polygene interaction rather than additive effect of the presence or absence of familial loading. On a neurocognitive level, one interpretation is that this increased activation represents a compensatory response or increased cognitive effort in those without a family history at the higher end of the schizophrenia risk spectrum, with a lack of such response in the familial group. This type of response has indeed been reported previously, where increased frontal activation occurs in response to increased task cognitive load (Manoach, 2003). Genetic origins of the interaction findings could be attributable to a number of factors. Firstly, epistatic effects, whereby the effects of the contributing SNPs are modified by genetic background related to the presence of positive family history. Genetic risk factors not captured by the PGRS (including risk of less common or rare causal variants) could also be influencing the relationship. Also, the fact that the familial group was unaffected may mean that resilience factors may be present in a proportion of these individuals. Similarly, the contamination of shared environmental effects may also have an impact in the familial group. To further clarify this finding it would be important to examine effects in independent samples as well as in patient populations.

These results suggest that lateral prefrontal dysfunction is a heritable vulnerability factor for schizophrenia rather than a secondary consequence of illness or medication. The next step is to develop approaches that will provide a greater understanding of underlying aetiological processes, to provide strategies for treatment and illness prediction. SNPs included in the current study were derived from the PGC cross-disorder consortia where previous analysis of expression quantitative trait loci (eQTL) in post-mortem tissue indeed suggested enrichment for brain markers (Smoller et al., 2013). The current study indicates that there are also specific neurophysiological responses associated with these SNPs. In addition, previous pathway analysis from the cross-disorder group implicated significant enrichment of calcium channel signalling genes, implicating a specific biological pathway in the pathogenesis of these disorders (Smoller et al., 2013).

One important limitation is the potential bias towards schizophrenia studies, mentioned above. This finding could reflect the greater number of schizophrenia cases contributing to the original cross-disorder PGC data and hence greater power to detect an effect. However, the consistency with the schizophrenia literature would indicate that this finding has firm biological validity. Also to note is that we did not report any significant relationships between the PGRS and positive symptom scores from the PANSS, however, this is likely due to the fact that these were all currently well individuals with a limited range of scores. Another potential limitation relates to the generalisability of this study in the fact that individuals were recruited based on the presence of a positive family history. Whilst it is arguably possible that the findings may be less generalisable to sporadic cases, there is little empirical evidence that is the case. Indeed, for disorders where there is partial genetic penetrance along with a complex architecture, sporadic cases are to be expected and these individuals may be mechanistically very similar to those that arise from within multiplex families.

In summary we report association between cross-disorder PGRS and frontal brain activation in healthy individuals. This was in the absence of family history rather than a generalised effect across all individuals, and was associated specifically with the contribution of schizophrenia risk to the cross-disorder PGRS. This regional association is consistent with the notion that frontal cortical inefficiency is a circumscribed phenotype for schizophrenia, and suggests that neuroimaging deficits in frontal regions seen in other diagnostic groups may be related to the cross-over of schizophrenia risk seen in these disorders.

## Role of the funding source

HCW is supported by a Dorothy Hodgkin Fellowship from the Royal Society (DH080018). JES is supported by a Clinical Research Training Fellowship from the Wellcome Trust (08772/Z/08/Z). AMM is supported by the Health Foundation through a Clinician Scientist Fellowship (Ref: 2268/4295) and by a NARSAD independent investigator award. We gratefully acknowledge the support of the Theresa and Mortimer Sackler Foundation.

This study was conducted at the Scottish Brain Research Imaging Centre (www.bric.ed.ac.uk) which is supported by SINAPSE (Scottish Imaging Network, a Platform for Scientific Excellence, www.sinapse.ac.uk). The investigators also acknowledge the financial support of the National Health Service (NHS) Research Scotland, through the Scottish Mental Health Research Network (www.smhrn.org.uk) who provided assistance with subject recruitment and cognitive assessments.

## Contributors

HCW and AMM designed the current study. HCW wrote the first draft of the manuscript. JES and AMcd collected the functional and clinical data. LR, JES and HCW analysed the functional data. LH conducted the polygenic analysis. AMM and SML advised on all aspects of analysis. All authors contributed to and approved the final manuscript.

## Conflict of interest

SML and AMM have done consultancy work for Roche Pharmaceuticals. SML has received grants from AbbVie and Pfizer, and personal fees from Janssen. We report no other conflicts of interest.

## Figures and Tables

**Table 1:** Participant details.

	Controls (n = 57)		Bipolar high-risk (n = 82)		t/Z	p
Demographics						
Mean age (years) (std dev)	20.81	(2.33)	21.16	(2.78)	0.79	0.43
Gender (M:F)	28:29	–	45:37	–	0.67^a^	0.51
Mean NART IQ (std dev)	109.63	(7.40)	109.21	(8.66)	0.30	0.76

Clinical measures^b^ (median (range))						
bPANSS positive total	7	(3)	7	(4)	2.34	0.02
bPANSS negative total	7	(5)	7	(4)	0.20	0.85
bPANSS general total	16	(4)	17	(9)	1.58	0.14
bYMRS score	0	(4)	0	(3)	0.59	0.56
bHDRS score	0	(7)	0.5	(15)	2.38	0.02

PGRS						
Cross disorder	0.1634	(0.01)	0.1995	(0.10)	2.02	0.05
ADHD	0.4664	(0.36)	0.4152	(0.36)	0.82	0.42
AUT	− 0.1389	(0.06)	− 0.1377	(0.06)	0.16	0.87
BD	1.5292	(0.25)	1.6131	(0.27)	1.86	0.06
MDD	0.2801	(0.21)	0.3117	(0.21)	0.89	0.38
SCZ	− 0.3670	(0.20)	− 0.2934	(0.18)	2.20	0.03

Behavioural measures						
Word appropriateness scores	3.06	(0.54)	2.92	(0.56)	1.33	0.18
Reaction time	2492	(610)	2544	(647)	0.43	0.64

PANSS = positive and negative syndrome scale, YMRS = Young mania rating scale, HRDS = Hamilton depression rating scale, PGRS = polygenic risk score.

a Chi squared test.

b Mann–Whitney tests, median and range presented for skewed variables.

**Table 2:** Correlation coefficients between individual diagnoses and peak activation.

Diagnosis	Polygenic score with peak activation (r (p value))	Controlling for the other disorders (r (p value))	Test of difference of correlation coefficients with those for SCZ (Z score (p value))
*Control group*			
ADHD	0.10 *(p = 0.34)*	0.05 *(p = 0.73)*	1.64 (p = 0.05)
Autism	0.01 *(p = 0.60)*	0.03 *(p = 0.86)*	2.12 (p = 0.02)
Bipolar disorder	− 0.01 *(p = 1.00)*	− 0.01 *(p = 0.94)*	2.09 (p = 0.02)
Major Depressive Disorder	0.09 *(p = 0.82)*	0.08 *(p = 0.57)*	1.69 (p = 0.04)
Schizophrenia	0.38 **(p < 0.01)**	0.39 **(p < 0.01)**	n/a

*Familial group*			
ADHD	0.15 *(p = 0.16)*	0.18 *(p = 0.12)*	–
Autism	− 0.15 *(p = 0.19)*	− 0.19 *(p = 0.10)*	–
Bipolar disorder	− 0.06 *(p = 0.57)*	− 0.08 *(p = 0.51)*	–
Major Depressive Disorder	− 0.06 *(p = 0.60)*	− 0.07 *(p = 0.53)*	–
Schizophrenia	− 0.12 (p = 0.32)	− 0.11 (p = 0.34)	–

N.B.: using predictive utility differences (|r|).
