# Supplementary material for: Impact of cross-disorder polygenic risk on frontal brain activation with specific effect of schizophrenia risk
Source: Schizophr Res. 2015 Feb;161(2-3):484–9. doi: 10.1016/j.schres.2014.10.046 (PMC4396692; doi:10.1016/j.schres.2014.10.046)
Supplement: Supplementary file 1 — Supplementary material. [file mmc1.doc]

**Supplementary Material**

### Scanning procedure

Imaging was carried out at the Brain Imaging Research Centre (BIRC) for Scotland on a GE 1.5 T Signa scanner (GE Medical, Milwaukee, USA). The functional imaging protocol consisted of axial gradient-echo planar images (EPI) (TR/TE = 2000/40 ms; matrix = 64 x 64; field of view (fov) = 24 cm) acquired continually during the experimental paradigm. Twenty-seven contiguous 5 mm slices were acquired within each TR. Each EPI acquisition was run for 404 volumes the first four of which were discarded. The T1 sequence yielded 180 contiguous 1.2 mm coronal slices (matrix = 192 x 192; fov = 24 cm; flip angle = 8°). Visual stimuli were presented using a screen (IFIS, MRI Devices, Waukesha, WI, USA) placed in the bore of the magnet.

**Genotyping and derivation of polygenic scores**

PGC data, numbers for each diagnostic category (from Smollet er al., 1012): For autism these comprised of 4788 trio cases, 4788 trio pseudocontrols, 161 cases, 526 controls, for ADHD 1947 trio cases,1947 trio pseudocontrols, 840 cases, 688 controls, for BD 6990 cases, 4820 controls, MDD 9227 cases, 7383 controls, and SCZ 9379 cases, 7736 controls.

SNPs were excluded if they had a minor allele frequency (MAF) < 5%, deviated significantly from Hardy-Weinberg equilibrium (p<0.001) in the total sample of founder individuals, or had a call rate <98%. Individuals that had an overall SNP call rate of <98% were excluded from further analysis. Strand-ambiguous SNPs were also removed. Clump based linkage disequilibrium pruning (r^2^ 0.2, 300kb window) was performed to create a SNP-set in linkage equilibrium. Profile scores were generated by taking the sum of the PGC reference alleles weighted by the logarithm of odds ratio and divided by the number of SNPs used in the SNP set.

**Supplementary Figure 1** *Main task-related activations
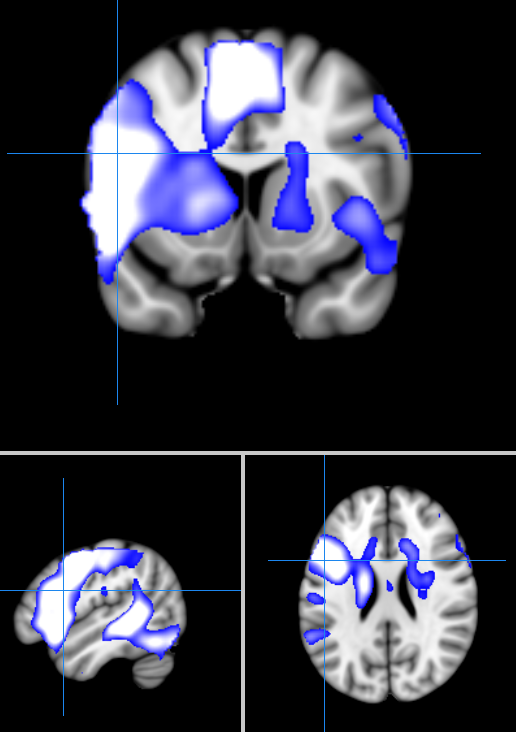
*

Depicts regions of activation for the contrast of [sentence completion versus baseline], demonstrating activation in lateral frontal cortex and lateral temporal cortex. Maps determined using within group random effects analysis of controls only. Images are overlaid onto standard brain in MNI space using Mango software package (<http://ric.uthscsa.edu/mango>). Map represents T-statistic images thresholded equivalent to p uncorrected=0.001.
